# New polyurethane prostheses for substitution of cardiac valve disease and remodeling of the right ventricle in congenital heart malformations

**DOI:** 10.1186/1749-8090-8-S1-O141

**Published:** 2013-09-11

**Authors:** MA Maluf

**Affiliations:** 1 Cardiovascular Division, São Paulo Federal University, Brazil

## Background

The biological cardiac prosthesis on the market today, are durable and functional, but still not the ideal valve replacement in children.

## Objectives

Develop three different models of polyurethane prostheses prototypes for substitution in cardiac valve disease and remodeling of the right ventricle, in patients with congenital heart disease.

## Methods

Based on a computed tomography angiography of the aorta, we made a delrin ring, keeping the anatomical characteristics of the aortic annulus. A matrix one following we made from stainless steel with the shape of the three cusp aortic valve, followed by preparation of the injection of liquid polyurethane segmented (SPU), using a esterolitografia technique. In vitro tests: The materials used in the manufacture of prostheses were approved in biocompatibility testing according to ISO 10993 - Biological evaluation of medical devices. The dentures will undergo physical tests, hydrodynamic and durability according to ISO 5840 - Cardiovascular Implants - Prosthetic heart valves.

## Results

The macroscopic appearance of these prototypes was approved by the group of Engineer, Biologist and Pediatric Cardiac Surgeon. It will be observed the macroscopic and microscopic optical and electronic scanning and imaging studies. The data will be sorted by making emphasis on the degree of calcification, presence of thrombi, infection and integrity of the prosthesis SPU. Experimental studies published showed good hemodynamic performance of the polyurethane prostheses, implanted on the right side of the heart: the absence of significant pressure gradients.

## Conclusions

It is possible to reproduce these results and further studies, carried out to better understand the properties of the SPU and level of reliability with a view on release, compared to health authorities for clinical application, thus becoming one more option among the prostheses on the market today.

